# Dosimetric characterization of Elekta stereotactic cones

**DOI:** 10.1002/acm2.12242

**Published:** 2017-12-20

**Authors:** Egor Borzov, Alexander Nevelsky, Raquel Bar‐Deroma, Itzhak Orion

**Affiliations:** ^1^ Department of Radiotherapy Division of Oncology Rambam Health Care Campus Haifa Israel; ^2^ Department of Nuclear Engineering Ben‐Gurion University of the Negev Beer Sheva Israel

**Keywords:** Elekta Versa HD, Monte Carlo simulation, small‐fields dosimetry, stereotactic cones

## Abstract

**Purpose:**

Dosimetry of small fields defined by stereotactic cones remains a challenging task. In this work, we report the results of commissioning measurements for the new Elekta stereotactic conical collimator system attached to the Elekta VersaHD linac and present the comparison between the measured and Monte Carlo (MC) calculated data for the 6 MV FFF beam. In addition, relative output factor (ROF) dependence on the stereotactic cone aperture variation was studied and penumbra comparison for small MLC‐based and cone‐based fields was performed.

**Methods:**

Cones with nominal diameters of 15 mm, 12.5 mm, 10 mm, 7.5 mm, and 5 mm were employed in our study. Percentage depth dose (PDD), off‐axis ratios (OAR), and ROF were measured using a stereotactic field diode (SFD). BEAMnrc code was used for MC simulations.

**Results:**

MC calculated and measured PDDs for all cones agreed within 1%/0.5 mm, and OAR profiles agreed within 1%/0.5 mm. ROF obtained from the measurements and MC calculations agreed within 2% for all cone sizes. Small‐field correction factors for the SFD detector K_field,3 × 3_(SFD) were derived using MC calculations as a baseline and were found to be 0.982, 0.992, 0.997, 1.015, and 1.017 for the 5, 7.5, 10, 12.5, and 15‐mm cones respectively. The difference in ROF was about 10%, 6%, 3.5%, 3%, 2.5%, and 2% for ±0.3 mm variations in 5, 7.5, 10, 12.5, and 15‐mm cone aperture respectively. In case of single static field, cone‐based collimation produced a sharper penumbra compared to the MLC‐based.

**Conclusions:**

Accurate MC simulation can be an effective tool for verification of dosimetric measurements of small fields. Due to the very high sensitivity of output factors on the cone diameter, manufacture‐related variations in cone size may lead to considerable variations in dosimetric characteristics of stereotactic cones.

## 1 INTRODUCTION

Stereotactic radiosurgery (SRS) is a noninvasive technique that delivers a single high dose of radiation to small, well‐defined intracranial lesions. To facilitate linear accelerator based SRS, modern linacs (Varian TrueBeam [Varian Medical Systems, Inc., Palo Alto, CA, USA] and Elekta VersaHD [Elekta AB, Stockholm, Sweden]) are equipped with high definition Multileaf Collimation System (MLC) and with a set of conical collimators, and offer the possibility of treatment with the flattening filter free (FFF) mode. SRS with MLC has been shown to be advantageous in most situations, since multiple isocenters are not required to obtain conformal dose distributions.^1^, ^2^, ^3^, ^4^ However, there are some cases (such as treatment of trigeminal neuralgia) where the use of MLC can be difficult due to: (a) possible MLC positional variations; (b) potential TPS dose calculation inaccuracy; and (c) high dose gradient required for sparing surrounding normal tissue. In addition, the use of MLC are not be optimal when targets are smaller than the leaf width.^5^, ^6^ In these situations, the use of cones with diameters of less than 1 cm can be preferable, because of: (a) higher mechanical stability; (b) the use in TPS of a single predefined dosimetric model for each cone; and (c) sharper dose fall‐off compared with the MLC because of the smaller source‐to‐collimator distance and focused cone aperture in contrast to the rounded shape of the MLC leaf ends.

Dosimetry of small fields defined by stereotactic cones remains a challenging task, mainly due to detector issues (position uncertainties, dose averaging, lack of electronic equilibrium, possibly tissue equivalence, etc.).^7^, ^8^ Therefore, the detector selected for the measurements must have a small active volume and high spatial resolution. There are several types of appropriate detectors: diamond detector, gel dosimeter, films, diode. The diode is one of the most frequently used detectors, but still has issues associated with energy, dose rate, and directional dependence of its response.^9^

To overcome the difficulties associated with small field measurements, it has been suggested to employ Monte Carlo (MC) techniques.^10^ The use of MC simulations allows verification of measurements obtained during commissioning of stereotactic systems and studying of the dosimetric characteristics which cannot be measured directly.

There are number of publications on MC modeling of small fields for the Varian TrueBeam linac.^11^, ^12^ A limited number of publications exist on dosimetry of stereotactic cones manufactured by Varian, BrainLab and Elekta.^13^, ^14^ Only a few reports on the full MC modeling of the Elekta VersaHD linac can be found in the literature^15^ and, to the best of our knowledge, there are no publications on MC modeling of stereotactic cones used with this linac.

The purpose of this work was to report on the results of commissioning measurements performed for small fields defined by the new Elekta stereotactic conical collimator system attached to Elekta Versa HD linac, and to present the comparison between the measured and MC calculated data for the 6 MV FFF beam. In addition, relative output factor (ROF) dependence on the stereotactic cone apertures variation was studied and penumbra comparison for small MLC‐based and cone‐based fields was performed.

## 2 MATERIALS AND METHODS

### 2.A Linac MLC and stereotactic conical collimator system

**Figure 1:**
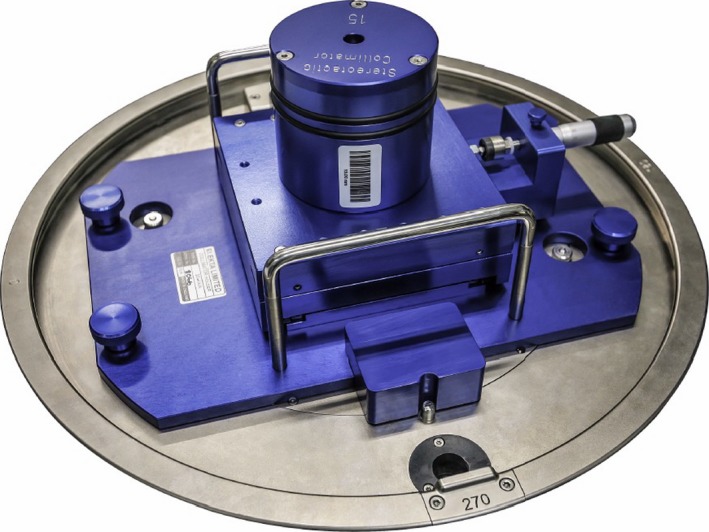
New Elekta's stereotactic collimation system: stereotactic cone and holder attached to the linac's head.

The Elekta VersaHD linac is equipped with the Agility MLC that has 160 leaves of projected 5 mm width at the isocenter.^16^ The new stereotactic circular cones are designed for the collimation of photon beams on a linear accelerator and are used as an additional accessory for it. The stereotactic conical collimation system consists of a collimator holder, which is attached to the linac's head, and a set of conical collimators (Fig. 1).

Two micrometers on the collimator holder are used to align the cone axis with the central axis of the beam. Conical collimators with nominal diameters of 15 mm, 12.5 mm, 10 mm, 7.5 mm, and 5 mm were employed in our study. When stereotactic cones are attached to the linac the field size defined by the MLC is set to 3 × 3 cm^2^ and kept constant for all cone sizes.

The stereotactic collimators have a conical opening in the center that is focused back to the radiation source in order to minimize the penumbra. The nominal diameter of the cone is defined as the projection of its opening at the isocenter. It is indicated on top of the cone and nominally corresponds to the value of the radiation field size measured at the 50% dose. The aperture accuracy of the fabricated cones is ±0.15 mm in diameter as stated in the drawings of the stereotactic cone system provided by the manufacturer, which corresponds to about ±0.22 mm variations at the isocenter. The actual cone size at the isocenter is measured during the installation and, according to the customer acceptance, test may differ from the nominal value up to 1 mm. In our study, the actual aperture diameter for each cone was found from lateral dose profiles measured in a water phantom.

Furthermore, in order to investigate ROF dependence on stereotactic cone aperture variations, the ROF for each cone was calculated for five aperture diameters: nominal size, nominal size ± 0.15 mm and nominal size ± 0.30 mm.

### 2.B Monte Carlo calculations

**Figure 2:**
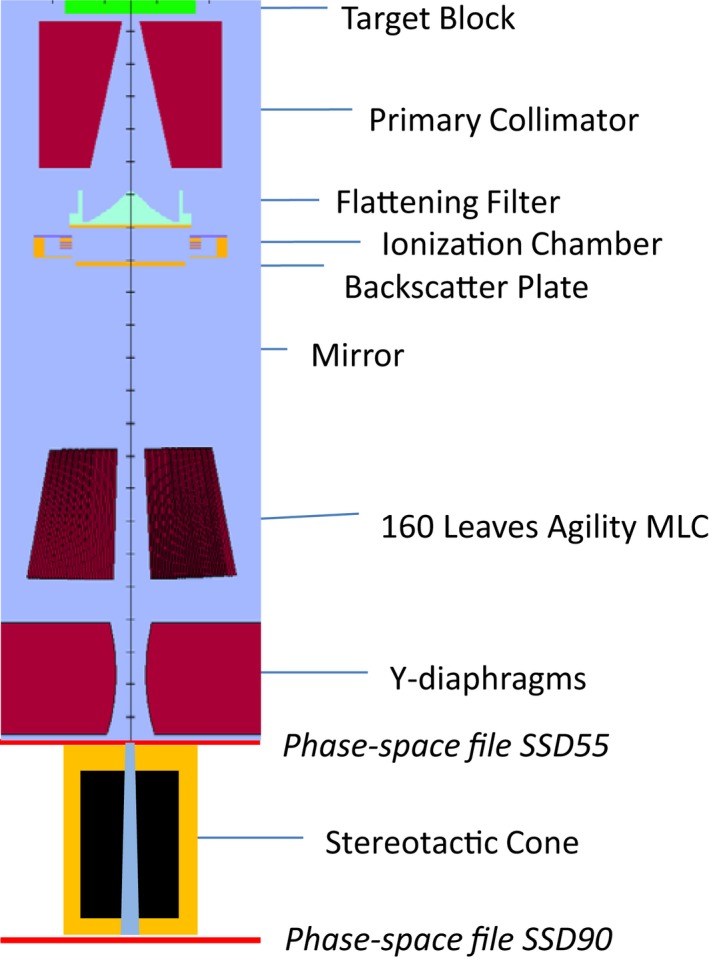
Modeling geometry of Elekta VersaHD head. Initial electron beam impact target block from the vacuum in the direction of Z‐axis. The component dimensions and material composition were provided by the manufacturer under nondisclosure agreement.

The Monte Carlo user code BEAMnrc^17^ was used to simulate transport through the accelerator head and cone applicator. The component modules were chosen to describe the following elements: target block with target insert, primary collimator, flattening filter (in FF mode only), ion chamber, backscatter plate, mirror, MLC, jaws (Y‐diaphragms in Elekta's terminology), mylar, and stereotactic circular collimator for cone fields only. The geometry model is shown in Fig. 2. To describe the Agility MLC, the component module MLCE was selected since it allows definition of the leaf bank rotation angle. The component dimensions and material composition were provided by the manufacturer under nondisclosure agreement.

To create a MC model of the linac head, the following parameters of the MLC and of the incident electron beam were determined by matching the measured and the calculated dose distributions: the incident beam spectrum, width and angular divergence, leaf bank rotation (LBROT) angle and leaf spacing at the isocenter. The incident beam spectrum was defined by matching the PDD for a 10 × 10 cm^2^ field, and the incident beam width was determined by matching the penumbra in both (in‐plane and cross‐plane) directions for a 2 × 2 cm^2^ field. The angular spread of the incident beam was adjusted by matching the profiles for a 20 × 20 cm^2^ field. LBROT angle and leaf spacing were obtained by matching the measured and calculated interleaf leakage.

To increase photon fluence efficiency, it was necessary to apply variance reduction techniques. Bremsstrahlung cross‐section enhancement (BCSE) is a variance reduction technique that was designed to increase the efficiency of simulations involving x‐ray production from bremsstrahlung targets. It was applied in our study for the linac target block media with an average enhancement factor of about 20. BCSE is compatible with other variance reduction techniques and is most efficient when used in conjunction with directional bremsstrahlung splitting (DBS). Following published recommendations^18^ DBS was used with a splitting number of 1000, a splitting radius of the field size value (e.g., 10 cm for a 10 × 10 cm^2^ field), the e+/e− splitting plane was set at the end of the backscatter plate and the Russian roulette plane was set slightly above the e+/e− splitting plane but still within the backscatter plate.

The DOSXYZnrc code was used to calculate the three‐dimensional absorbed radiation dose distributions in water. For each field size, a simulated phantom with adequate set of voxels was built. Voxels were created with sizes in the range of 0.5 × 0.5 × 1 mm^3^ for the cone‐based fields up to 4 × 4 × 10 mm^3^ for the 20 × 20 cm^2^ MLC‐based fields. Similar to BEAMnrc the cutoff energies P_cut_ and E_cut_ were set to 0.01 MeV and to 0.521–0.700 MeV respectively.^17^

PDD, OAR, and ROF were calculated with the DOSXYZnrc code. ROFs were defined at the depth of 10 cm as the ratio of dose D_field_ from a field of interest to the dose D_10 × 10_ from the reference field of 10 × 10 cm^2^:

(1) $${\mathrm{ROF}}_{\mathrm{MC}}=\frac{D_{\mathrm{field}}}{D_{10\times 10}}$$

The number of histories used in MC simulations was selected so that a statistical uncertainty of less than 1% in ROF calculations was achieved. ROFs calculated with MC simulations will be further designated as ROF_MC._

The dose backscattered to the monitor chamber in Elekta linacs was not accounted for and no corrections for field size dependence of monitor chamber dose were made during ROF calculations using MC simulations.

To investigate ROF dependence on stereotactic cone aperture variations, the ROF for each cone was calculated with MC simulations for five aperture diameters: nominal size, nominal size ± 0.15 mm and nominal size ± 0.30 mm.

MC simulations were also used to calculate the cone transmission factor defined as the ratio of the dose transmitted through the cone with completely blocked aperture to the dose from the reference 10 × 10 cm^2^ field.

### 2.C Measurements

To create a MC model of the linac, we used PDD, off‐axis ratios (OAR) and ROF for a set of MLC‐based square fields (1 × 1, 2 × 2, 3 × 3, 5 × 5, 10 × 10, and 20 × 20 cm^2^) measured during the linac commissioning for 6 MV and 6 MV FFF beams. Data acquired for commissioning of the conical collimators included measurements of PDD, OAR, and OF for each cone at SSD = 90 cm. In this work, the ROF obtained from the measurements is designated as ROF_meas_. The ROF_meas_ for cone‐based and MLC‐based fields were measured at a depth of 10 cm and were normalized to a reference field size of 10 × 10 cm^2^ :

(2) $${\mathrm{ROF}}_{\mathrm{meas}}=\frac{M_{\mathrm{field}}}{M_{10\times 10}}$$

where M is the detector reading.

The measurements were performed in a water phantom (MP3, PTW, Freiburg, Germany) using an ionization chamber (IC; PTW 31010, Semiflex) for fields 3 × 3 cm^2^ and larger and with stereotactic field diode (SFD; IBA Dosimetry, Schwarzenbruck, Germany) for fields 3 × 3 cm^2^ and smaller. In our study, ROFmeas for field sizes larger than 3 × 3 cm^2^ were defined as ratio of the ion chamber (IC) readings:

(3) $${\mathrm{ROF}}_{\mathrm{meas}}=\frac{M_{\mathrm{field}}\left(\mathrm{IC}\right)}{M_{10\times 10}\left(\mathrm{IC}\right)}$$

ROF_meas_ for fields less than 3 × 3 cm^2^ were determined by the so‐called “daisy‐chaining”^19^, ^20^ approach: first, the ratio of the output readings measured with SFD for a small field and the 3 × 3 cm^2^ field was calculated, and then it was renormalized by applying the ratio of output readings measured with the Semiflex ionization chamber for the 3 × 3 cm^2^ and the reference 10 × 10 cm^2^ field, according to the Eq. (4):

(4) $${\mathrm{ROF}}_{\mathrm{meas}}=\frac{M_{\mathrm{field}}\left(\mathrm{SFD}\right)}{M_{3\times 3}\left(\mathrm{SFD}\right)}\times \frac{M_{3\times 3}\left(\mathrm{IC}\right)}{M_{10\times 10}\left(\mathrm{IC}\right)}$$

To estimate uncertainty of the measured values, the data from Cranmer‐Sarginson et al.^21^ can be used. For all setup combinations involving fields less than 3 × 3 cm^2^, we expect the uncertainty in ROF_meas_ to be not greater than 0.5% except for the field from 5‐mm cone where uncertainty is expected to be around 1%. Uncertainty of ROFs measured with the ion chamber (for fields larger than 3 × 3 cm^2^) is estimated to be about 0.1% and can be assumed to be negligible.

As mentioned above, measurements for small fields may be subject to errors introduced by detector averaging effects, fluence perturbations caused by detector presence and spectral dependence of detector response. In an attempt to overcome the measurement related uncertainties, Alfonso et al.^19^ presented a methodology which makes use of MC calculated, detector‐specific, correction factor K. According to this methodology, to obtain ROF defined as the ratio of absorbed dose to water for the field of interest to that of the reference field (D_field_/D_ref_), one should multiply the ratio of the detector readings for the field of interest and reference field (M_field_/M_ref_) by the correction factor K_field,ref_. Using this formalism Eq. (4) can be rewritten as

(5) $${\mathrm{ROF}}_{\mathrm{field}}=\frac{M_{\mathrm{field}}\left(\mathrm{SFD}\right)}{M_{3\times 3}\left(\mathrm{SFD}\right)}\times \frac{M_{3\times 3}\left(\mathrm{IC}\right)}{M_{10\times 10}\left(\mathrm{IC}\right)}\times K_{\mathrm{field},3\times 3}\left(\mathrm{SFD}\right)\times K_{3\times 3,10\times 10}\left(\mathrm{IC}\right)$$

K_3 × 3,10 × 10_(IC) may be assumed to be close to unity since IC is small compared to the field size of 3 × 3 cm^2^ and is not influenced by spectral changes between 10 × 10 cm^2^ and 3 × 3 cm^2^ fields.^15^ Then Eq. (5) may be rewritten as

(6) $$\mathrm{ROF}\left(\mathrm{field}\right)={\mathrm{ROF}}_{\mathrm{meas}}\left(\mathrm{field}\right)\times K_{\mathrm{field},3\times 3}\left(\mathrm{SFD}\right)$$

For the smallest field size of 0.5 × 0.5 cm, K_0.5 × 0.5,3 × 3_(SFD) was shown to be between 0.966 and 1 according to several publications in which various treatment machines and various methods of calculation were employed.^7^, ^22^, ^23^, ^24^, ^25^ The reason for the range of the K values is that this factor, apart from being detector‐specific, is also energy spectrum specific and as such depends on equipment used and measurement conditions (SSD and depth). Moreover, It should be noted that no data on K_field,3 × 3_(SFD) have been reported before for the same equipment and the same measurement conditions as in our study (stereotactic cones attached to the VersaHD linac and used with 6 MV FFF beams). Therefore, we cannot fully implement the formalism of Alfonso and apply the correction factor K to the ratio of detector readings.

However, if ROF(field) is calculated directly using MC as the ratio of D_field_ and D_10 × 10_ and if MC calculations are considered as free of detector‐related errors, then one can calculate K_field,3 × 3_(SFD) from Eq. (6) as

(7) $$K_{\mathrm{field},3\times 3}\left(\mathrm{SFD}\right)=\frac{{\mathrm{ROF}}_{\mathrm{MC}}\left(\mathrm{field}\right)}{{\mathrm{ROF}}_{\mathrm{meas}}\left(\mathrm{field}\right)}$$

The uncertainty of K defined in this way combines the uncertainties of the measurement and MC calculations and is estimated to be 1.1% for all fields except for the field from 5‐mm cone where this uncertainty is 1.4%.

### 2.D Penumbra comparison for the cone‐based and MLC‐based fields

In order to perform penumbra comparison for the cone‐based and MLC‐based fields, penumbra width defined as the distance between the points representing 20% and 80% of the central axis dose (P20/80) was calculated for a static 1 × 1 cm^2^ square field and a 10‐mm cone field. However, characterization of penumbra is more clinically relevant when radiation is delivered through a full arc gantry rotation. Dose distributions for both static and rotational fields were calculated in a homogenous cylindrical water phantom with a radius of 11 cm created in DOSXYZnrc using the CTCREATE code. The axis of the cylindrical phantom was oriented along “Gantry‐Target” direction and SSD was 89 cm. The phase space files were placed so that the rotation isocenter was located in the middle of the cylinder. The DOSXYZnrc “Phase space source incident from multiple directions” option was used in order to simulate source rotation. In‐plane and cross‐plane profiles were calculated and corresponding P20/80 values were obtained.

## 3 RESULTS

Table 1 presents the summary of the MLC and the incident electron beam parameters used in the MC model of the 6 MV FFF beam. Parameters of the 6 MV flattened beam are shown as well for the sake of comparison.

**Table 1:** Summary of adjusted initial electron beam parameters

Parameter	FF mode	FFF mode
Mean energy, MeV	6.5	7.4
Energy FWHM, MeV	0.5	0.5
Electron beam width cross‐plane/in‐plane FWHM, mm	0.15/0.25	0.10/0.20
Mean angular speed, degrees	1.1	0.6
LBROT angle, rad	0.1	
Leaf spacing at iso, cm	0.5	

**Figure 3:**
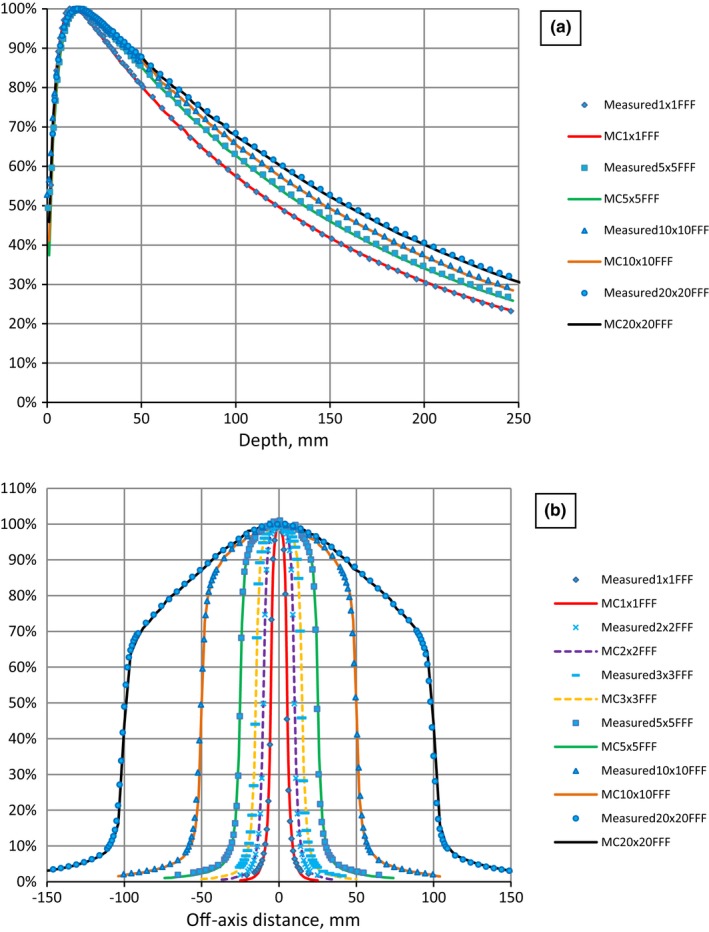
Measured and Monte Carlo modeled (MC) PDD (a) and profiles (b) for 6 MV FFF.

For square MLC‐based fields calculated and measured PDDs for all field sizes agreed within 1%/0.5 mm. OAR profiles in in‐plane and cross‐plane directions agreed within 1%/1 mm for all field sizes (Fig. 3).

**Figure 4:**
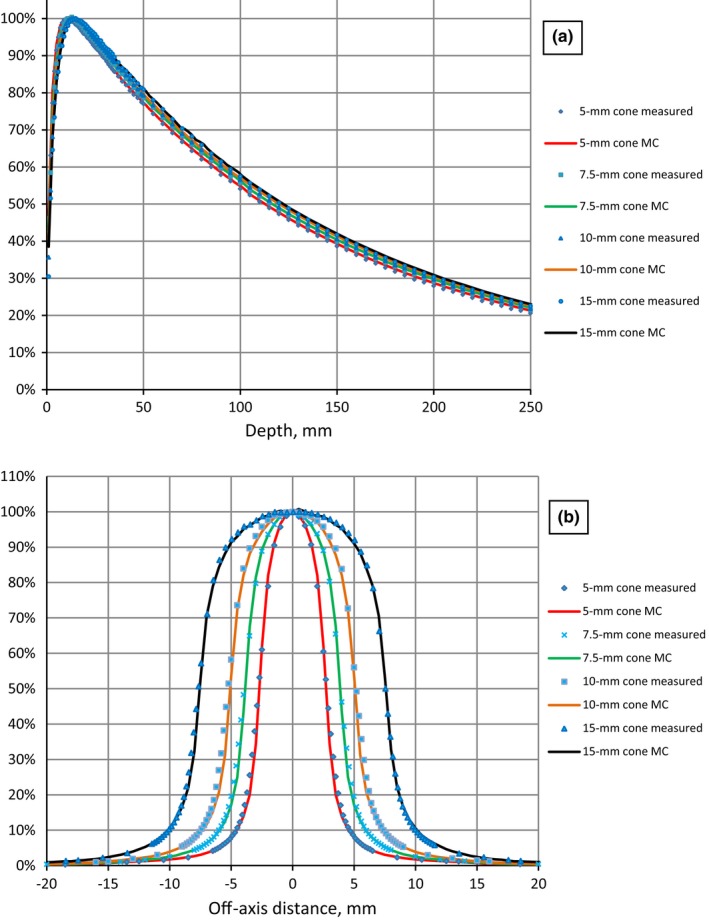
Measured and Monte Carlo modeled (MC) PDD (a) and profiles (b) of stereotactic cones for 6 MV FFF.

For circular cone‐based fields calculated and measured PDDs for all cones are in agreement within 1%/0.5 mm, OAR profiles (after aperture size adjustment) agreed within 1%/0.5 mm (Fig. 4).

The results of ROF_meas_ and ROF_MC_ comparison are presented in Table 2. The difference does not exceed 1.0% for all fields.

**Table 2:** Measured and modeled relative output factors for 6 MV FFF square fields

Field size	Measured ROF	MC ROF
1 × 1	0.711	0.713
2 × 2	0.825	0.825
3 × 3	0.880	0.876
5 × 5	0.928	0.920
10 × 10	1	1
20 × 20	1.060	1.054

**Figure 5:**
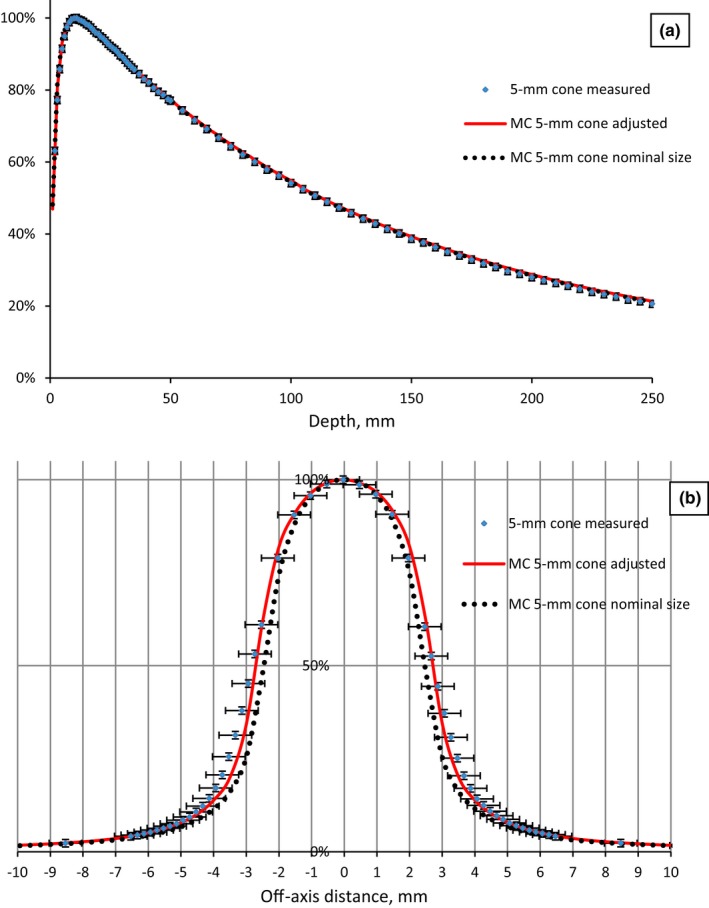
PDD (a) and profiles (b) for 5‐mm cone: measured data, MC data for nominal size, and MC adjusted data. Measured data with (±1%, ±0.5 mm) error bars.

Figure 5 shows calculated OAR profile for nominal cone diameter of 5 mm and for adjusted diameter, and the measured OAR profile. The influence of this aperture adjustment on the PDD shape was not observed.

For all cones Table 3 presents aperture adjustment, ROF_meas_, ROF_MC_ for adjusted cone size, and calculated K factors.

**Table 3:** Aperture adjustment for each cone, ROF measured data and ROF calculated with MC for adjusted cone size

Nominal cone size, mm	Aperture adjustment, mm	ROF measured	ROF MC calculated	K_field,3 × 3_ (SFD)
5	+ 0.3	0.564	0.554	0.982 ± 0.014
7.5	+ 0.2	0.648	0.643	0.992 ± 0.011
10	+ 0.15	0.706	0.704	0.997 ± 0.011
12.5	+ 0.15	0.740	0.751	1.015 ± 0.011
15	+ 0.15	0.770	0.782	1.017 ± 0.011

**Figure 6:**
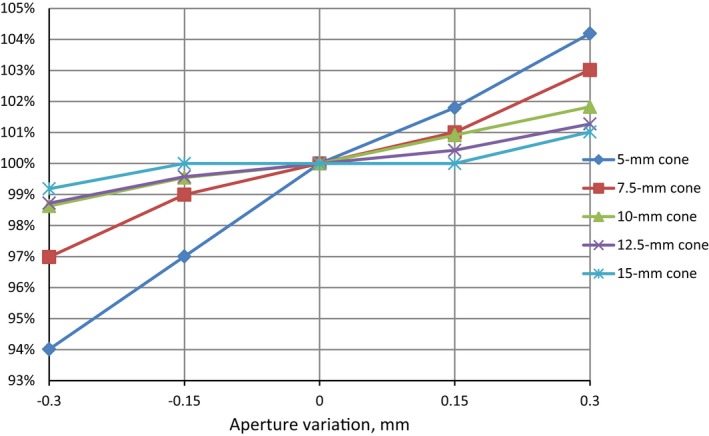
ROF as function of aperture variation, normalized for each cone at the value of ROF at the nominal cone size.

Dependence of ROF on aperture size variations for each cone is shown in Fig. 6, which presents ROF as function of aperture variation, normalized for each cone at the value of ROF at the nominal cone size.

**Figure 7:**
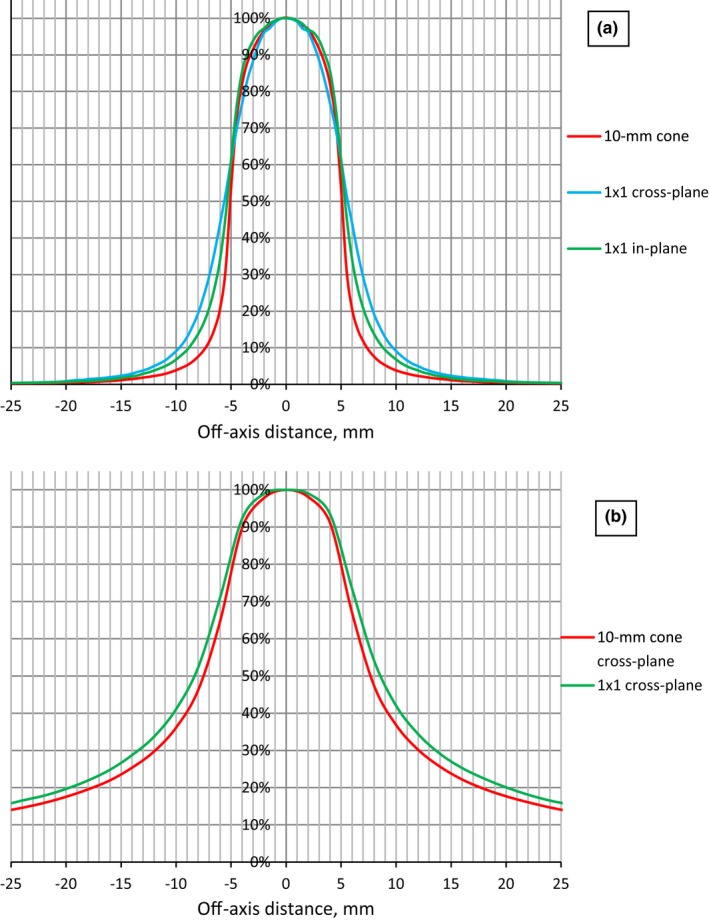
Comparison of 10‐mm cone profile and 1 × 1 square field profiles at isocenter. (a) Static fields in a cylindrical phantom; (b) Rotational fields in a cylindrical phantom.

Figure 7(a) presents calculated profiles for static fields in a cylindrical phantom. Penumbra width for a 1 × 1 cm^2^ field is 2.8 mm and 4.2 mm for cross‐plane and in‐plane, respectively, and 2.0 mm for both cone 10 mm profiles. Results of calculated cross‐plane profiles for rotational fields in a cylindrical water phantom are shown on Fig. 7(b). Penumbra width is 13.0 mm for cone and 14.8 mm for square field. For in‐plane profiles the values remained the same as for static fields, as expected.

Cone transmission factor was found to be 0.5%.

## 4 DISCUSSION

Characteristics of X‐ray dose distributions from linear accelerators have been shown to be very sensitive to the parameters of the incident electron beam, such as energy, spectral and spatial distribution, and beam divergence.^26^ In our study, the electron source was modeled as an elliptical beam with Gaussian distribution in in‐plane and cross‐plane, nonzero angular spread and Gaussian energy distribution. The elliptical shape of the electron source in Elekta linacs is well‐known^27^ and partially explains different penumbra widths in in‐plane and cross‐plane directions. Apart from the electron source shape, the penumbra of the radiation field is strongly affected by the geometry of the collimation system (MLC and jaws). Therefore, while for square fields the penumbra in the cross‐plane direction is wider than in the in‐plane direction, for the cone‐based fields the in‐plane and cross‐plane profiles are identical, because the cones are positioned much closer to the detector. Leaf‐bank rotation of 0.01 radian is a manufacturer's invention aimed at reducing interleaf leakage.^28^ Parameters adjustment were made for FF and FFF modes separately. Parameters of the MC model for the 6 MV beam found in our work are very similar to those reported in a previous study of the Agility MLC.^15^ Simulation results are in good agreement with measured PDD, profiles and output factors for square and cone fields.

All measured cone diameters were found to be systematically larger than the corresponding nominal sizes in the range 0.15 to 0.30 mm. ROF obtained from the measurements (ROF_meas_) and from the MC calculations (ROF_MC_) agreed within 2% for all cone sizes. Assuming that the difference between ROF_meas_ and ROF_MC_ can be fully attributed to the measurement errors caused by the presence of the SFD diode, correction factors K_field,3 × 3_(SFD) could be calculated and were found to be 0.982, 0.992, 0.997, 1.015, and 1.017 for the 5, 7.5, 10, 12.5, and 15‐mm cones respectively.

The high sensitivity of relative output factors to small changes in cone size was demonstrated. Clearly the greatest sensitivity is observed for the smallest cone. One can also see that for the 5‐mm cone, negative aperture variations (producing smaller aperture size) cause greater ROF changes than positive variations. This effect can be due to the increased source occlusion.^10^ The difference in ROF was about 10%, 6%, 3.5%, 3%, 2.5%, and 2% for ± 0.3 mm variations in 5, 7.5, 10, 12.5, and 15‐mm cone aperture respectively. Taking into account the cone manufacturing accuracy, the measured output factors for the same nominal cone size may differ between medical centers.

Such sensitivity of ROF to changes in field aperture is typical for small field size, whether it is formed by MLC or by cones. However, cone aperture is not expected to change after being commissioned, unlike MLC‐based fields. Therefore, one can see the advantage in using cones for very small field sizes since they are less prone to ROF variations.

As dose backscattered to the ion chamber may change by several percents with field size, some authors recommended proper simulation of this dose as part of the MC calculations.^24^ In Elekta linacs, however, there is a backscatter plate which prevents particles from the downstream direction entering the monitor chamber. It was shown^29^ that with the backscatter plate in place the proportion of particles backscattered into the monitor chamber is less than 0.35% and, therefore, corrections for field size dependence in monitor chamber dose are not necessary when running MC simulations of the Elekta linac. Moreover, since jaw opening is kept constant for all cone sizes, backscattering into the monitor chamber will not influence the calculation of ROF for stereotactic cones.

The cone leakage was calculated according to manufacturer's measurement. Our result of 0.5% is less than the measured 0.65% and can be caused by different host machines settings.

The penumbra of a single static field defined by 10‐mm cone was sharper than the penumbra of 1 × 1 cm^2^ square field defined by MLC, especially in the cross‐plane direction. However, when full arc irradiation with 10‐mm cone and 1 × 1 cm^2^ square is considered, the cross‐plane penumbra becomes comparable for both collimations and the advantage of the stereotactic cone almost disappears, while for in‐plane profiles the values remained the same as for static fields. Additional analysis based on clinical cases for practical volumes of PTV and organs of risk irradiated with several noncoplanar arcs is needed.

## 5 CONCLUSIONS

The results of our MC calculations were found to be in good agreement with the measurements. Due to the very high sensitivity of output factors on the cone diameter, manufacture‐related variations in cone size may lead to considerable variations in dosimetric characteristics of stereotactic cones. Therefore, no “gold data” sets are possible for cones of the same nominal diameter. K_field,3 × 3_(SFD) factors are suggested for our equipment and our measurement geometry. For one static field, cone‐based collimation produces a sharper penumbra compared to the MLC‐based collimation; however, this advantage becomes negligible for cross‐plane penumbra in case of rotational field. Accurate MC simulation can be an effective tool for verification of dosimetric measurements such as ROF of small fields.

## CONFLICT OF INTEREST

No author has any conflict of interest.
